# Decreased expression of *MT1E* is a potential biomarker of prostate cancer progression

**DOI:** 10.18632/oncotarget.18683

**Published:** 2017-06-27

**Authors:** Rita Demidenko, Kristina Daniunaite, Arnas Bakavicius, Rasa Sabaliauskaite, Aiste Skeberdyte, Donatas Petroska, Arvydas Laurinavicius, Feliksas Jankevicius, Juozas R. Lazutka, Sonata Jarmalaite

**Affiliations:** ^1^ Life Sciences Centre, Vilnius University, Vilnius, Lithuania; ^2^ National Cancer Institute, Vilnius, Lithuania; ^3^ Urology Centre, Vilnius University, Vilnius, Lithuania; ^4^ National Centre of Pathology, Affiliate of Vilnius University Hospital Santaros Klinikos, Vilnius, Lithuania; ^5^ Faculty of Medicine, Vilnius University, Vilnius, Lithuania

**Keywords:** prostate cancer, biochemical recurrence, gene expression, *MT1E*

## Abstract

Differentiation of indolent and aggressive prostate carcinoma (PCa) at the time of diagnosis is currently one of the major challenges. This study aimed at identification of prognostic biomarkers to aid in predicting biochemical recurrence (BCR) of the disease. Microarray-based gene expression profiling in tissues of 8 BCR and 8 No-BCR cases revealed expression differences of 455 genes, most of which were down-regulated in BCR cases. Eleven genes were selected for validation by real-time PCR in the first PCa cohort (N = 55), while seven of them were further validated in the second, independent, PCa cohort (N = 53). Down-regulation of *MT1E* (p < 0.001) and *GPR52* (p = 0.002) expression and up-regulated levels of *EZH2* (p = 0.025) were specific biomarkers of BCR in at least one of the two PCa cohorts, but only *MT1E* expression retained the independent prognostic value in a multivariate analysis (p < 0.001). DNA methylation analysis (114 PCa and 24 non-cancerous tissues) showed frequent *MT1E* methylation in PCa (p < 0.001) and was associated (p < 0.010) with the down-regulated expression in one PCa cohort. The results of our study suggest *MT1E* down-regulation as a potential feature of aggressive PCa.

## INTRODUCTION

Prostate carcinoma (PCa) is the second most common cancer and the fifth leading cause of death from cancer among men according to GLOBOCAN 2012 [1]. Implementation of prostate-specific antigen (PSA) testing into clinical practice led to marked increase in the numbers of newly diagnosed early stage PCa. However, even localized PCa is a highly heterogeneous disease with variable clinical outcomes ranging from indolent, low-risk disease to tumors with aggressive behavior having a tendency to progress. The risk evaluation based on D’Amico’s classification system (clinical stage, PSA level, and histological features of the tumor on biopsy) is also not informative enough, with nearly one half of cases being upgraded/upstaged at final pathology [2]. Molecular markers may provide more accurate measures of tumor aggressiveness and assist in the discrimination between indolent forms of PCa and clinically aggressive tumors.

During recent years, several molecular changes were identified as PCa-specific biomarkers, while potent prognostic biomarkers for early detection of aggressive forms of PCa are still lacking. In combination with other biomarkers, the fusion of androgen-regulated gene *TMPRSS2* with *ERG* (*TMPRSS2-ERG*) or other genes of the oncogenic ETS transcription factor family may assist in outcome prediction, but has no independent prognostic value [3, 4–6]. Over-expression of *SPINK1* is detectable in a subset of ERG-negative cases and can mark an aggressive course of the disease [7, 8]. However, the prognostic value of ERG or SPINK1 was not confirmed in a study of large multi-institutional cohort of early stage PCa cases [9]. Recent studies have also suggested the prognostic value of several other genetic alterations, including over-expression of the *EZH2* gene [10, 11]. However, taking into consideration that multiple genomic alterations in prostate tumors may define patients with different cancer risks, further studies of novel molecular markers in large independent cohorts are needed to improve our understanding of PCa behavior and to provide clinicians with more precise indicators of aggressive course of the disease.

In the present study, genome-wide gene expression profile in tissues of PCa cases that experienced biochemical recurrence (BCR) was compared to non-recurrent cases utilizing microarray technology. A total of 11 gene targets were chosen from microarray analysis or selected from publications [6, 12] and thoroughly validated in two independent cohorts of PCa cases by means of real-time PCR. Possible involvement of DNA methylation in expression changes of the most significantly down-regulated gene *MT1E* was analyzed in a subset of PCa cases and controls.

## RESULTS

### Global gene expression profiling

**Figure 1:**
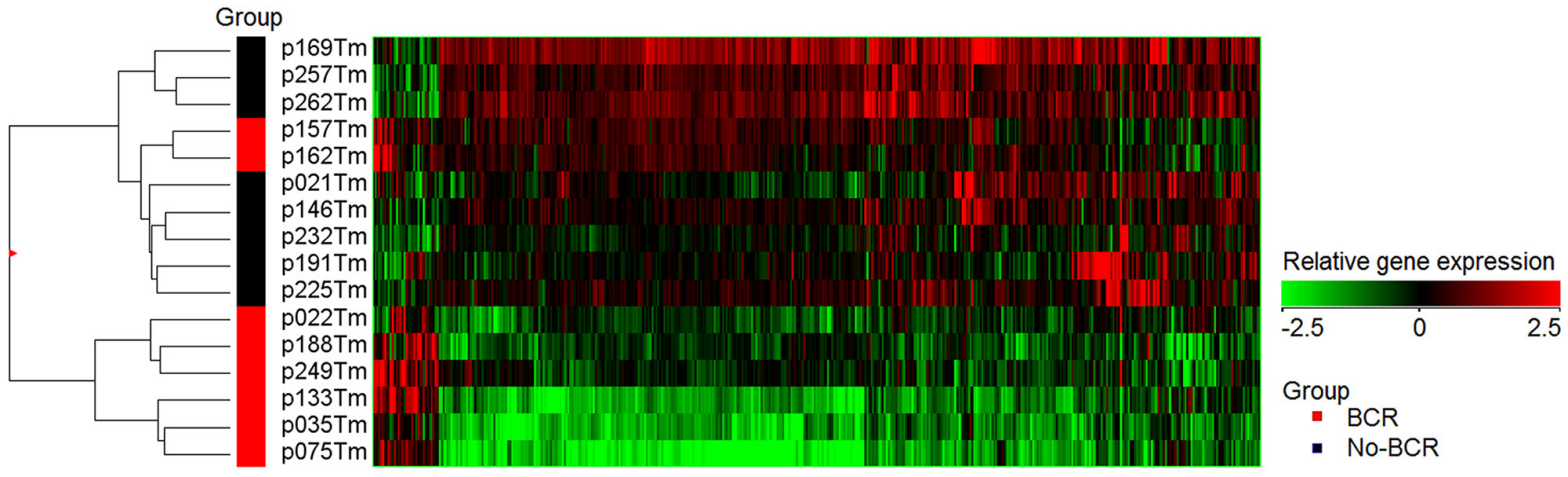
Heatmap of gene expression in prostate tissue Expression profile of the genes that were differentially expressed (N = 455, p < 0.050) in biochemically recurrent (BCR) and No-BCR cases.

Microarray-based transcriptome comparison of 8 BCR and 8 No-BCR PCa samples identified 455 genes that were significantly deregulated (p < 0.050) with absolute fold change (FC) value of ≥2 (Figure 1). The vast majority of these genes were down-regulated (421 of 455; 92.5%) and only 34 (7.5%) were up-regulated in BCR cases as compared to No-BCR. Among the genes showing the most significant decrease of expression were *GPR52* (FC 2.3; p < 0.001), *GHRH* (FC 2.2; p < 0.001), and *FABP7* (FC 3.05; p = 0.009), whereas *CHI3L2* (FC 3.6; p = 0.002), *SAA2* (FC 6.2; p = 0.014), *OLR1* (FC 2.0; p = 0.005) were significantly over-expressed in BCR cases. Comparing PCa cases with early (≤6 mo; N = 4) and late (>6 mo; N = 4) BCR, significant down-regulation of *MT1E* was detected (FC 3.0; p = 0.026).

### Data validation in PCa1 cohort

In order to explore the prognostic value of the novel biomarkers, seven genes (*CHI3L2, FABP7, GHRH, GPR52, MT1E, OLR1,* and *SAA2)* significantly deregulated in the microarray screening were selected for further validation on custom design TaqMan Low Density Array (TLDA) cards using cancerous (N = 55) and non-cancerous (N = 12) prostate tissue (NPT) samples from PCa1 cohort. In addition, several well-known PCa biomarkers (*ERG, EZH2, HPN,* and *TERT*) with prognostic value shown in our previous study and in studies of other authors [6, 12] were also included into this analysis.

In PCa1 cohort, comparison of PCa to NPT samples, revealed significant up-regulation of *HPN* (FC 3.3; p < 0.001), *ERG* (FC 3.0; p = 0.038), and *EZH2* (FC 1.3; p = 0.029) in cancerous tissues, while *FABP7* (FC 4.4; p < 0.001) was significantly down-regulated (Supplementary Figure 1A). Other significant changes of gene expression in various clinical sub-groups are presented in Supplementary Figure 1A.

**Figure 2:**
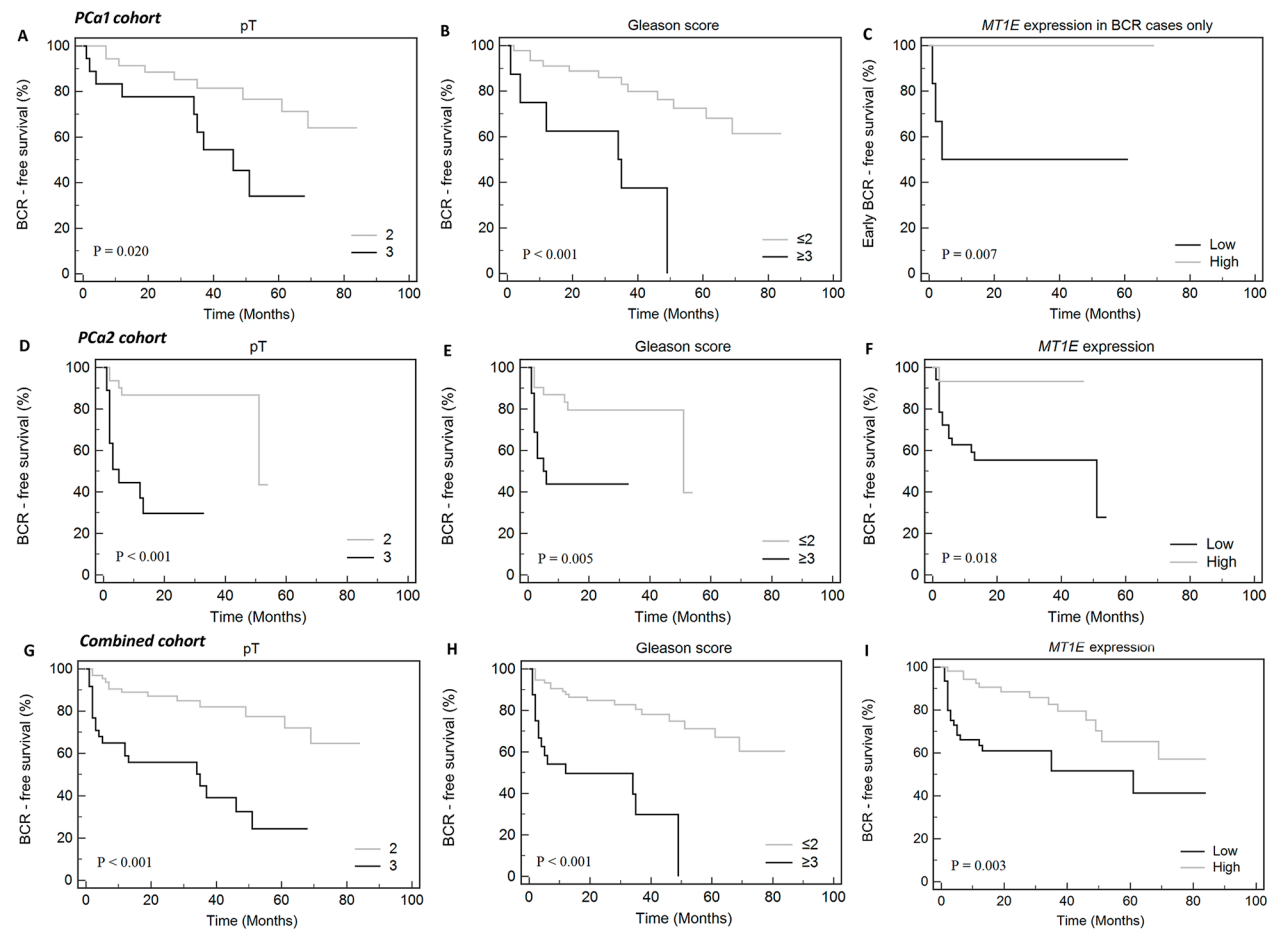
Kaplan-Meier curves of biochemical recurrence-free (BCR-free) survival according to clinical-pathological variables and *MT1E* expression level **(A and B)** – PCa1 cohort, **(C)** – PCa1 cohort, only cases with BCR, dichotomized defining early BCR (≤6 mo) as a complete event and late BCR (>6 mo) as a censored event, **(D-F)** – PCa2 cohort, **(G-I)** – combined cohort (PCa1 & PCa2). Only parameters significantly associated with BCR or early BCR are shown (p < 0.050).

Comparison of BCR to No-BCR cases (N = 18 and N = 35, respectively) revealed significant down-regulation of *MT1E* (FC 2.0; p < 0.001) and *GPR52* (FC 1.7; p = 0.002) in accordance with the disease progression (Supplementary Figure 1A). In Kaplan-Meier analysis, *GPR52* (p = 0.033), in addition to tumor stage (pT; p = 0.020) and grade group (G; p < 0.001), was a significant predictor of BCR (Figure 2A, 2B). Moreover, lower *MT1E* expression level was predictive of the early disease progression (≤6 mo; p = 0.007) (Figure 2C). In univariate Cox regression hazard analysis, clinical variables – G and pT – were significant predictors of BCR, while reduced expression of the *MT1E* gene in PCa1 cohort showed no significance (p = 0.070; Supplementary Table 1).

### Data validation in PCa2 cohort

The prognostic value of a set of genes showing significant associations with BCR and other clinical variables in PCa1 study (*ERG*, *EZH2*, *GPR52*, *HPN*, *MT1E*, *OLR1*, and *TERT*) was further analyzed in an independent cohort of 53 PCa cases utilizing TaqMan-based single-assay real-time PCR. Similarly to the PCa1 cohort, comparison of BCR to No-BCR cases (N = 16 and N = 33) revealed significant decrease of *MT1E* expression (FC 3.8, p < 0.001). In PCa2, BCR was also associated with higher levels of *EZH2* (FC 1.4, p = 0.025; Supplementary Figure 1B). Kaplan-Meier curves revealed pT (p < 0.001) and G (p = 0.005) together with *MT1E* (p = 0.018) expression as significant predictors of BCR (Figure 2D–2F). These findings were supported by univariate Cox regression hazard analysis (Supplementary Table 1). Moreover, *EZH2* expression level was also predictive of BCR in univariate Cox model (Supplementary Table 1), although Kaplan-Meier analysis showed no significance (data not shown).

### Combined gene expression analysis in both cohorts

**Figure 3:**
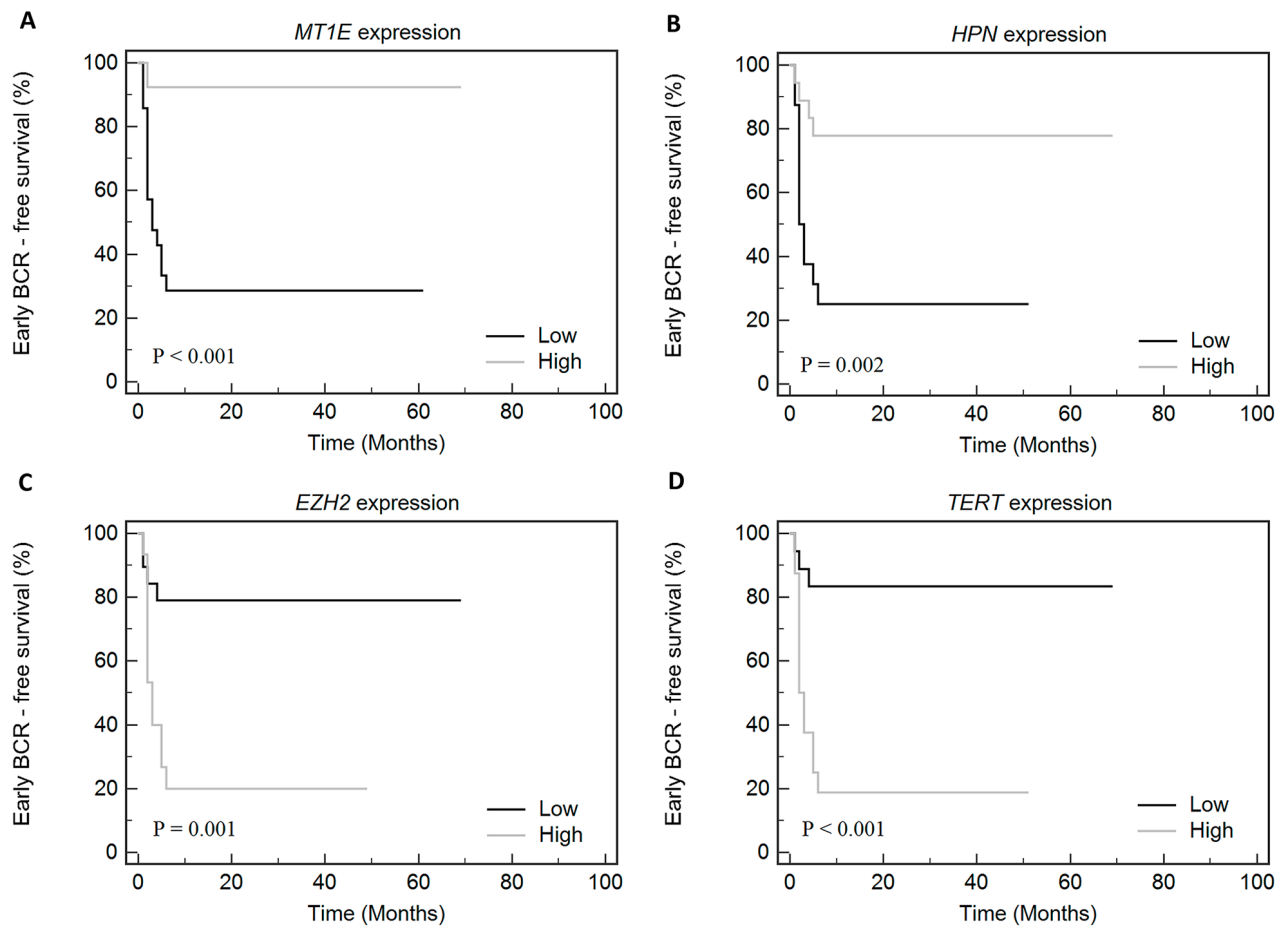
Kaplan-Meier curves according to the gene ((A) - *MT1E*, (B) - *HPN*, (C) - *EZH2*, (D) - *TERT*) expression levels in the combined cohort of cases with early (≤6 mo) and late (>6 mo) biochemical recurrence (BCR)

To summarize our study, survival analysis was performed for the combined group of PCa cases (N = 102 in total; 34 BCR and 68 No-BCR cases). Kaplan-Meier analysis revealed that BCR-free survival was significantly impacted by pT (p < 0.001), G (p < 0.001), and *MT1E* expression (p = 0.003; Figure 2G–2I), while *GPR52* expression showed a borderline significance (p = 0.057). In BCR group, early progression was significantly associated with low *MT1E* and *HPN* expression levels and high *EZH2* and *TERT* levels (Figure 3A–3D).

In the multivariate Cox regression model, backward entering of covariates revealed significant predictive value of pT, G, and *MT1E* expression (model’s p < 0.001). Out of the gene expression data evaluated, only *MT1E* expression level maintained an independent predictive value for the BCR in multivariate analysis (p < 0.001; Table 1).

**Table 1:** Cox regression analysis for clinical-pathological parameters and biomarkers in combined PCa1 and PCa2 cohort

Parameter	Univariate			Multivariate		
	Hazard ratio [95% CI]	P-value	Model’s P-value	Hazard ratio [95% CI]	P-value	Model’s P-value
Tumor stage (pT3 vs pT2)	4.28 [2.11; 8.65]	**<0.001**	**<0.001**	2.81 [1.34; 5.88]	**0.006**	**<0.001**
ISUP Gleason grading group	5.15 [2.48; 10.70]	**<0.001**	**<0.001**	2.67 [1.21; 5.91]	**0.015**	
Preoperative PSA	1.02 [1.00; 1.04]	0.062	0.106	backward eliminated		
*MT1E* expression	0.59 [0.48; 0.72]	**<0.001**	<0.001	0.68 [0.55; 0.85]	**0.001**	
*GPR52* expression	0.78 [0.54; 1.13]	0.186	0.187	backward eliminated		
*EZH2* expression	1.66 [1.19; 2.31]	**0.003**	**0.003**	backward eliminated		
*OLR1* expression	1.21 [1.04; 1.40]	**0.013**	**0.012**	backward eliminated		
*HPN* expression	0.76 [0.59; 0.98]	**0.031**	**0.031**	backward eliminated		

Significant P-values are in bold ISUP – International Society of Urological Pathology, PSA – prostate-specific antigen, CI – confidence interval.

Significant associations between clinical variables and between clinical variables and genes’ expression are provided in Supplementary Table 2.

### DNA methylation analysis of *MT1E*

**Figure 4:**
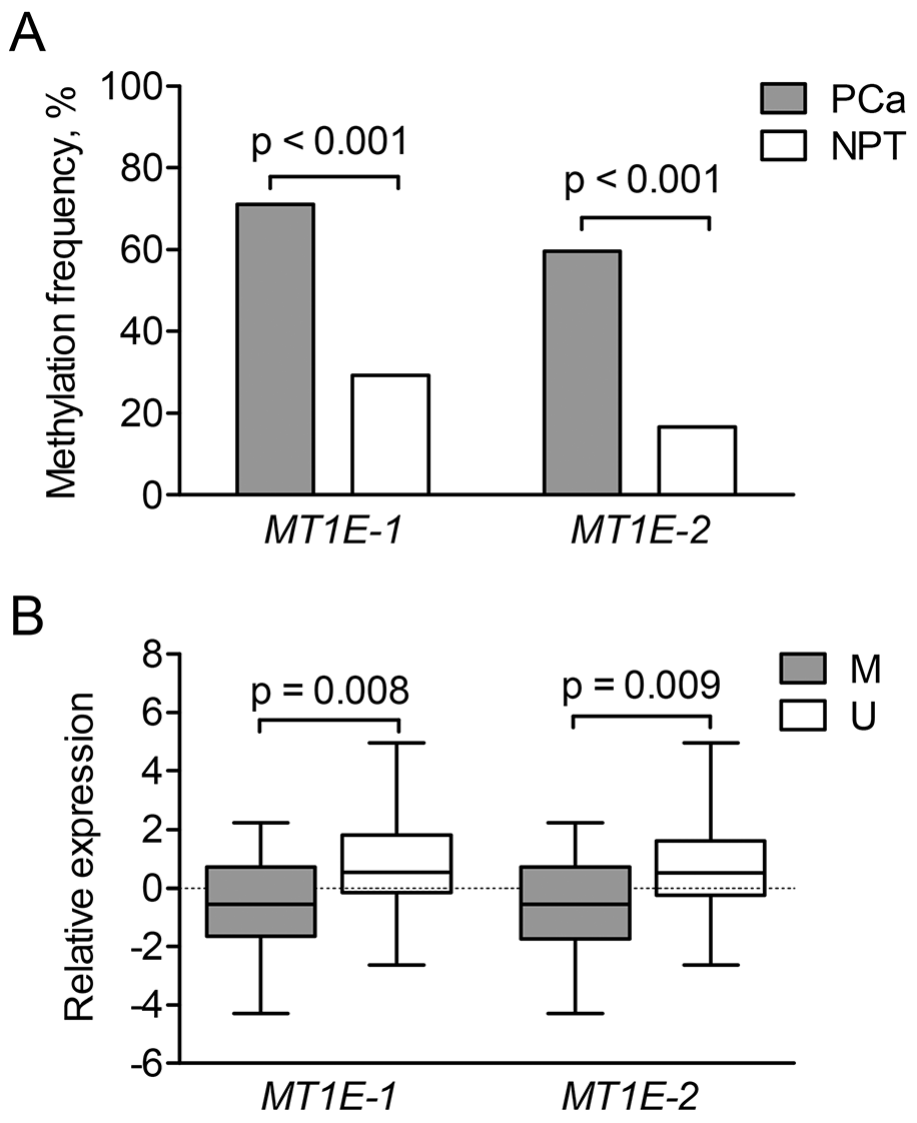
*MT1E* methylation analysis **(A)** – Aberrant methylation frequencies of the two *MT1E* gene promoter-associated fragments (*MT1E-1* and *MT1E-2*) in the MSP group. **(B)** – Relative expression of the *MT1E* gene according to the methylation status of the two analyzed fragments in tumors of PCa2 cohort. Gene expression values were mean-centered separately for each cohort and represented as median (line) with 25-75^th^ percentiles (box) and range (whiskers). PCa – prostate cancer, NPT – non-cancerous prostate tissue M/ U - methylated/ unmethylated promoter status..

To elucidate the mechanism of *MT1E* down-regulation in PCa, two *MT1E* fragments (*MT1E-1* and *MT1E-2*; Supplementary Figure 2) were analyzed by means of methylation-specific PCR (MSP) in most of the cases from PCa1 and PCa2 cohorts (114 PCa and 24 NPT, overall). Aberrant methylation was frequent in PCa and significantly less common in NPT samples (p < 0.001 and p < 0.001 for *MT1E-1* and *MT1E-2*, respectively) (Figure 4A). Both *MT1E-1* and *MT1E-2* fragments were more frequently methylated in pT3 than pT2 tumors (p = 0.029 and p = 0.009, respectively), but showed no other associations with clinical-pathological parameters. Methylated status of *MT1E-1* or *MT1E-2* fragments was significantly associated with lower *MT1E* gene expression level in tumors of PCa2 cohort (p = 0.008 and p = 0.009, respectively; Figure 4B), but no such association was observed in PCa1 tumors.

## DISCUSSION

During recent years, combinations of molecular biomarkers with the PSA test have suggested some promises for improved detection of PCa, however, their prognostic power remains questionable [10, 13]. Our study aimed at detection of PCa biomarkers prognostic for BCR after the surgery. Genome-wide gene expression profiling in BCR and No-BCR cases with up to 7 years of clinical follow-up data led to the identification of 455 differentially expressed genes. After the validation in two independent PCa cohorts with different duration of follow-up, down-regulation of *MT1E* was determined as a potential biomarker of early BCR onset in PCa.

Metallothioneins (MTs) are a group of metal binding proteins involved in the intracellular turnover of metal ions and detoxification of heavy metals [14]. The role of MTs has been extensively studied in PCa, but other genes from this superfamily (*MT1G*, *MT1H*, *MT3*) have attracted more attention than *MT1E* [15–17]. In general, down-regulation of MTs is considered to be an early step of cancer progression, associated with poor prognosis in different cancer patients [14, 18, 19]. In agreement with this, the down-regulation of *MT1E* expression was strongly associated with BCR in the two PCa cohorts in our study, regardless of the variation in the duration of follow-up. Besides, *MT1E* down-regulation was predominant in the cases with early BCR and showed associations with clinical markers of poor prognosis, i.e. higher grade group and more advanced tumor stage. In addition, our study revealed DNA methylation of the promoter region of the *MT1E* gene as a possible mechanism of gene inactivation in PCa. The aberrant *MT1E* promoter methylation was frequently observed in PCa tissues, but almost never occurred in NPT. *MT1E* promoter hypermethylation was previously reported only in melanoma and endometrial cancer patients [20, 21], but, to the best of our knowledge, has never been assessed in PCa.

Up-regulation of EZH2 (Enhancer of zeste homolog 2), a subunit of Polycomb repressive complex 2 (PRC2) with a histone methyltransferase activity, is recorded as one of the most common genetic alterations of PCa [22]. Elevated *EZH2* gene expression has been associated with metastatic PCa and aggressive course of the disease in clinically localized PCa cases, and independent prognostic value of this biomarker has been demonstrated in several studies [10, 11, 23]. Based on this data, the gene was included in our study and showed significant associations with BCR in one of our cohorts. In addition to the prognostic value of *EZH2*, a higher expression level of the gene was demonstrated in cancerous than non-cancerous tissues from patients with PCa, showing the potential diagnostic value of this biomarker.

Several biomarkers have been suggested for the stratification of localized PCa into high and low-risk disease and, therefore, could help clinicians in decision making and appropriate treatment selection. Our previous study [6] revealed the prognostic value of a combined *TMPRSS2-ERG* and *TERT* expression analysis, while the data of other authors [10, 11, 23] and our present study suggested up-regulation of *EZH2* and down-regulation of *MT1E* as the possible biomarkers of poor prognosis in PCa. Although PCa-specific transcripts are easily detectable in tumors, they are less abundant in blood or urine used for noninvasive monitoring of disease progression after therapy. The data of the present study suggest involvement of promoter DNA methylation as a putative mechanism that might be responsible for the *MT1E* down-regulation in PCa. The possibility to detect hypermethylated *MT1E* in body fluids of PCa patients, primarily focusing on evaluating its prognostic potential, remains to be explored in future studies.

In conclusion, our study indicates that reduced expression of*MT1E* is an independent predictor of poor prognosis in PCa. Measurement of *MT1E* level, along with other indicators of the disease progression, may serve for better prognostic stratification of PCa at the time of diagnosis or surgery.

## MATERIALS AND METHODS

### Tissue samples

Prostate tissue samples were obtained from PSA-screened and biopsy-proven PCa patients treated with radical prostatectomy (RP) at the Urology Centre of Vilnius University Hospital Santaros Clinics during the period 2008-2014. Approval from the Lithuanian Bioethics Committee was obtained for the study and all patients signed informed consent for participation.

The samples, collected during separate periods, composed two independent cohorts: group PCa1 in 2008-2010 and group PCa2 in 2010-2014 (Table 2). Cancerous (≥50% of tumor cells) and non-cancerous (0%) prostatectomy tissues were sampled by expert pathologist as previously reported [6] and prepared for molecular analysis. Gleason score was obtained according to International Society of Urological Pathology (ISUP) 2005 and 2014 recommendations [24]. Grade groups formed according to ISUP 2014 recommendations were merged into two clusters G ≤ 2 and G ≥ 3. In agreement with the EAU guidelines [25], BCR after RP was defined by two consecutive PSA values of ≥0.2 ng/mL and rising.

**Table 2:** Clinical-pathological parameters of the study groups.

Variable	Group PCa1^a^		Group PCa2^b^		MSP group^c^	Combined PCa1 & PCa2 group
	PCa (N = 55)	NPT (N = 12)	PCa (N = 53)	NPT (N = 24)	PCa (N = 114)	PCa (N = 108)
Mean age, years	62.1	61.5	58.4	61.5	60.5	60.3
Tumor stage, N						
pT2	36		34		75	70
pT3	19		19		39	38
ISUP Gleason grading group, N						
G≤2	47		36		92	83
G≥3	8		17		22	25
BCR, N						
Yes	18		16		32	34
No	35		33		74	68
Unknown	2		4		8	6
Mean follow-up time, months						
Mean	51		28		32	41
Median	54		27		29.5	37
Range	[1; 84]		[1; 72]		[1; 84]	[1; 84]
*TMPRSS2-ERG* status, N						
Positive	36		36		76	72
Negative	19		15		36	34
Unknown	0		2		2	2
Mean preoperative PSA level, ng/ml	11.46		10.18		10.89	10.82

BCR – biochemical recurrence; ISUP – International Society of Urological Pathology, MSP – methylation-specific PCR, NPT – non-cancerous prostate tissue, PCa – cancerous prostate tissue, PSA – prostate-specific antigen.

a – Samples were used for gene expression analysis by custom design TaqMan Low Density Arrays (TLDA).

b – Samples were used for gene expression analysis by TaqMan single assays.

c – Samples were used for *MT1E* gene promoter methylation analysis by MSP.

PCa1 cohort (55 PCa and 12 NPT samples) had a long follow-up period (mean 51 mo after RP) with a follow-up data collected for 96% (53/55) of the cases (Table 2). Based on the BCR status, PCa samples from this cohort were selected for microarray-based gene expression profiling and were also used for the data validation on custom design TLDA cards. PCa2 cohort, comprised of PCa cases with the shorter follow-up period (mean 28 mo), was used for the independent data validation by TaqMan Gene Expression Assays analysis. Follow-up data were collected for 92% (49/53) of PCa2 cohort patients (Table 2). Most of the PCa1 and PCa2 samples and some additional cases and controls (114 PCa and 24 NPT samples in total; Table 2) were assessed for aberrant DNA methylation.

### RNA and DNA preparation

Total RNA from snap-frozen sections, ground to powder using liquid nitrogen, was isolated with mirVana Kit (Ambion, Thermo Fisher Scientific, Foster City, CA, USA) according to the manufacturer’s recommendations and used for TLDA and single-gene expression assays. Quantity of the RNA samples was measured spectrophotometrically using the NanoDrop 2000 (Thermo Fisher Scientific, Wilmington, NC, USA). Integrity of the RNA samples (RIN) was assessed with 2100 Bioanalyzer (Agilent Technologies, Santa Clara, CA, USA) prior to analyses, and values ranged up to 9.7 (mean 8.0). Samples having RIN ≥7 were included in the study cohorts.

DNA was extracted from 10-30 mg of homogenized prostate tissues. Samples were digested with proteinase-K (Thermo Fisher Scientific, Vilnius, Lithuania) for up to 16 hours, then purified according to standard phenol-chloroform protocol followed by ethanol precipitation. For methylation analysis, 400 ng of extracted DNA were modified with sodium bisulfite using EZ DNA Methylation Kit (Zymo Research, Irvine, CA, USA) according to manufacturer’s protocol.

### Global gene expression profiling

Global gene expression profiling (GEO accession identifier GSE89317) of PCa tissues from 8 BCR and 8 No-BCR cases was performed onto SurePrint G3 Human Gene Expression (v2) 8×60 K microarrays (design ID 028004; Agilent Technologies). Sample processing (100 ng of input RNA) was performed according to One-Color Microarray-Based Gene Expression Analysis Low Input Quick Amp Labeling, version 6.5, protocol using spike RNA (RNA Spike-In kit) and Low Input Quick Amp Labeling Kit, One-Color (Agilent Technologies). Samples were hybridized at 65°C for 17 hours. Then, slides were washed using Gene Expression Wash Buffer kit and scanned with SureScan microarray scanner (Agilent Technologies).

Signal intensities from the images were extracted and evaluated with Feature Extraction software v10.7.3 and further analyzed using GeneSpring GX v12.6.0 (Agilent Technologies). Probes with saturated, non-uniform, and outlier signal values were removed before further preprocessing. Data was normalized according to 75th percentile and the median of all samples was used for the baseline transformation. Probes having raw signal values of ≤10 in more than 50% of the analyzed samples were filtered out. Probe annotations were extracted from eArray platform according to the corresponding microarray design identifier. Fold change (FC) values were estimated and unpaired t-test was used for two group comparison. Differences of gene expression levels were considered significant if absolute FC was ≥2.0 and p-value was <0.050.

### Gene expression analysis using TLDA arrays

Gene expression levels of 11 selected target genes and 5 reference genes (Supplementary Table 3) were quantified in triplicates using custom TLDA (Applied Biosystems, Thermo Fisher Scientific, Foster City, CA, USA) in samples from PCa1 cohort. Five hundred ng of total RNA were reverse transcribed (RT) to cDNA using High Capacity cDNA Reverse Transcription Kit with RNase Inhibitor (Applied Biosystems) according to the manufacturer’s instructions. Twenty μL of cDNA (equivalent to 500 ng of total RNA in each port) was immediately used as a template in real-time PCR (RT-qPCR).

### Gene expression analysis using TaqMan gene expression assays

Gene expression levels of 7 genes and 2 reference genes were analyzed with TaqMan gene expression assays (Supplementary Table 3) in PCa2 cohort. Only genes expressed in at least 70% of the samples from PCa1 were included in this step.

Genomic DNA from the extracted RNA samples was removed by digesting with 1 unit of DNaseI (Thermo Fisher Scientific, Vilnius, Lithuania). For the RT, 300 ng of total RNA were converted to cDNA using Maxima First Strand cDNA Synthesis Kit for RT-qPCR (ThermoFisher Scientific, Vilnius, Lithuania) in a final volume of 20 μL under the following conditions: 25°C for 10 min, followed by 50°C for 30 min, and 85°C for 5 min. Two μL of cDNA mix were used as a template in a total 20 μL qPCR volume.

RT-qPCR was performed using the TaqMan Universal Master Mix II no UNG following manufacturer’s recommendations (Applied Biosystems). Expression levels of all genes for all samples were analyzed in triplicates and required at least 2 valid wells. Non-template controls were included for each gene in each RT-qPCR run.

### DNA methylation analysis

Bisulfite-modified DNA was used as a template for MSP. Two sets of primers covering the promoter region of the *MT1E* gene (positioned at -112/+105 and -239/-91 from transcription start site, *MT1E-1* and *MT1E-2*, respectively) were designed with Methyl Primer Express software v1.0 (Applied Biosystems) and ordered from Metabion (Martinsried, Germany). The reaction mix (25 μL) contained 1x Maxima Hot Start Taq PCR buffer, 2.5 mM MgCl_2_, 1.6 mM dNTP mix, 1.25 U Maxima Hot Start Taq DNA polymerase (Thermo Fisher Scientific, Vilnius, Lithuania), 1 μM of each primer, and 1 μL of bisulfite-modified DNA. MSP reaction conditions included 37-38 cycles with primer annealing step at 55-56°C for 45 s. Bisulfite-modified leukocyte DNA from healthy donors served as a negative control for methylated DNA and SssI methyltransferase-treated (Thermo Fisher Scientific, Vilnius, Lithuania) bisulfite-modified leukocyte DNA served as a positive control. Non-template controls were included in each MSP run.

### RT-qPCR data processing and statistical analysis

Raw Cq-values were calculated using the Viia7 version 1.1 software (Applied Biosystems), applying automatically selected thresholds. According to NormFinder and GeNorm algorithms, the combination of *HPRT1, GUSB,* and *GAPDH* was shown as the most suitable set of reference genes in TLDA analyses, while the *HPRT1* and *GUSB* genes were used for single assays’ data normalization.

Computation of statistical tests was performed using GraphPad Prism version 5.00 for Windows (GraphPad Software, San Diego, CA, USA) and GenEx version 6.0.1 software systems (MultiD Analyses AB, Göteborg, Sweden). Survival analysis was carried out with MedCalc Statistical Software version 14.12.0 (MedCalc Software, Ostend, Belgium). Continuous variables were checked for normal distribution by Shapiro-Wilk statistic and compared by Student’s t-test when normally distributed or by the Mann-Whitney U test for non-normally distributed variables. Spearman’s rank correlation coefficient was calculated to test the associations between two variables with non-normal distribution. Pearson’s Chi-squared test and Fisher’s exact t-test were used for comparison of categorical variables. For survival analysis, Kaplan-Meier curves were compared using Log-rank (Mantel-Cox) test, and Cox proportional-hazards model, applying backward algorithm, was used for multivariate analysis. For regression analysis, gene expression levels were categorized as “high” or “low” if the log-transformed values were above or below the mean value of all samples for a particular gene, respectively. P-value < 0.050 was considered statistically significant.

## SUPPLEMENTARY MATERIALS FIGURES AND TABLES



## Abbreviations

BCR: Biochemical recurrence
G: Gleason grade group according to ISUP
ISUP: International Society of Urological Pathology
MSP: Methylation-specific PCR
NPT: Non-cancerous prostate tissues
PCa: Prostate carcinoma
PSA: Prostate-specific antigen
pT: Tumor stage
RP: Radical prostatectomy
TLDA: TaqMan low density array
