# Supplementary material for: Decreased expression of MT1E is a potential biomarker of prostate cancer progression
Source: Oncotarget. 2017 Jun 27;8(37):61709–18. doi: 10.18632/oncotarget.18683 (PMC5617458; doi:10.18632/oncotarget.18683)
Supplement: Supplementary file 1 [file oncotarget-08-61709-s001.pdf]

Decreased expression of *MT1E* is a potential biomarker of prostate cancer progression

SUPPLEMENTARY MATERIALS

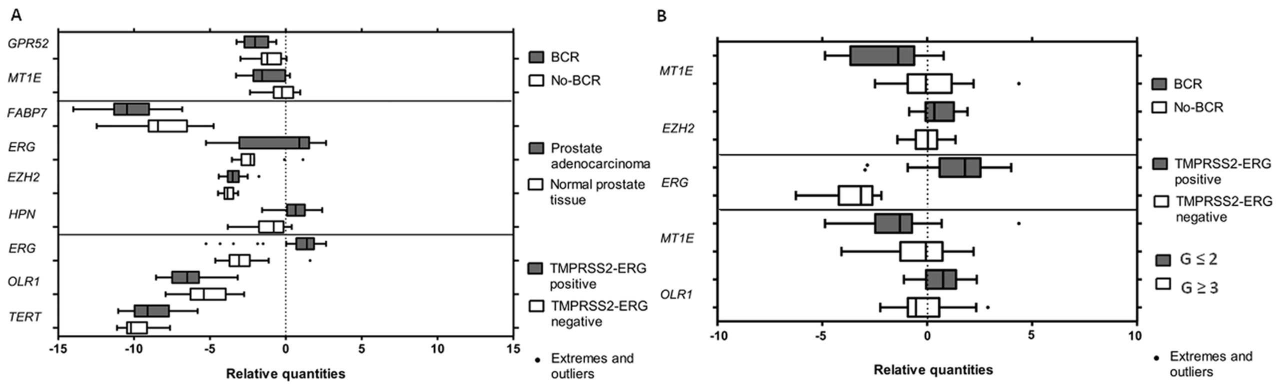

Supplementary Figure 1: Genes showing significantly different expression levels between groups in TLDA (A) and single gene assay RT-qPCR (B) experiment analyses. BCR – biochemical recurrence, No-BCR – non-relapsed cases, *TMPRSS2-ERG* positive/negative – *TMPRRS2-ERG* fusion-positive/negative prostate cancer cases.

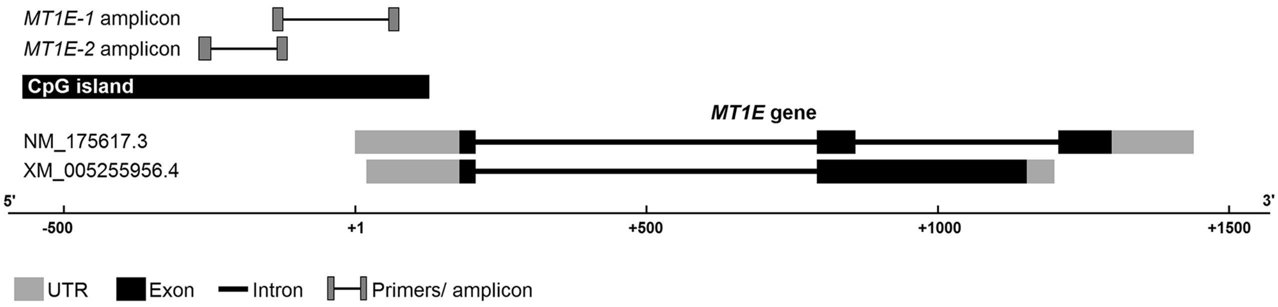

Supplementary Figure 2: Schematic representation of the two fragments (*MT1E-1* and *MT1E-2*), amplified with primers for methylation-specific PCR (MSP), with regard to *MT1E* gene location. Two known *MT1E* gene transcripts are shown. Transcription start site of the protein-coding transcript (NM_175617.3) is depicted as +1. Sequences for the illustration were obtained from GeneBank. UTR – untranslated region.

Supplementary Table 1: Cox regression hazard analysis in PCa1 and PCa2 cohorts

**PCa1 cohort**	**Univariate Cox**	**Significance**	
**Parameter**	**Hazard ratio [95% CI]**	**P-value**	**Model P-value**
Tumor stage (pT3 vs pT2)	2.9469 [1.1285 - 7.6954]	**0.0273**	**0.0292**
ISUP Gleason grade group (G3-5 vs G1-2)	5.0014 [1.7631 - 14.1875]	**0.0025**	**0.0056**
Preoperative PSA	0.9975 [0.9528 - 1.0443]	0.9147	0.9130
MT1E expression[a]	0.6643 [0.4265 - 1.0346]	0.0704	0.0747
GPR52 expression	0.7059 [0.4272 - 1.1664]	0.1741	0.1725
EZH2 expression	1.9683 [0.7451 - 5.1994]	0.1718	0.1728
HPN expression	0.7928 [0.4615 - 1.3620]	0.4004	0.4073
**PCa2 cohort**	**Univariate Cox**	**Significance**	
**Parameter**	**Hazard ratio [95% CI]**	**P-value**	**Model P-value**
Tumor stage (pT3 vs pT2)	7.4605 [2.3523 - 23.6616]	**0.0006**	**0.0002**
ISUP Gleason grading group	3.8448 [1.3616 - 10.8566]	**0.0110**	**0.0103**
Preoperative PSA	1.0705 [1.0296 - 1.1130]	**0.0006**	**0.0020**
MT1E expression	0.5471 [0.4020 - 0.7445]	**0.0001**	**0.0001**
GPR52 expression	1.0011 [0.5611 - 1.7862]	0.9969	0.9952
EZH2 expression	2.393 [1.1685 - 4.9003]	**0.0170**	**0.0167**
HPN expression	0.7504 [0.3898 - 1.4445]	0.3901	0.3900

Significant P-values are in bold.

[a]For gene expression, normalized Ct values were used.

Supplementary Table 2: Significant associations between clinical variables, and between clinical variables and genes' expression in combined cohort

Mann-Whitney U-test	Z	P-level	N
pT & PSA	3.225	0.001	106
pT & *MT1E*	-2.792	0.005	108
G & PSA	2.154	0.031	106
G & *MT1E*	-3.500	0.000	108
G & *OLR1*	3.340	0.001	108
G & *EZH2*	2.997	0.003	108
BCR & PSA	2.092	0.036	100
BCR & *MT1E*	-3.464	0.001	102
TMPRSS2-ERG & ERG	7.039	<0.001	106
Spearman's correlation	R	P-level	N
ERG & HPN	0.339	<0.001	108
EZH2 & HPN	-0.592	<0.001	108
GPR52 & MT1E	0.429	<0.001	108
MT1E & EZH2	-0.488	<0.001	108
MT1E & HPN	0.367	<0.001	108
MT1E & OLR1	-0.523	<0.001	108
MT1E & TERT	-0.469	<0.001	108
OLR1 & ERG	-0.305	<0.001	108
OLR1 & EZH2	0.802	<0.001	108
OLR1 & HPN	-0.702	<0.001	108
OLR1 & TERT	0.744	<0.001	108
TERT & EZH2	0.868	<0.001	108
TERT & HPN	-0.624	<0.001	108
Fisher's exact test		P-level	N
G & BCR		0.001	102
pT & BCR		0.001	102
pT & G		0.004	108

pT – tumor stage, G – ISUP grade group, BCR – biochemical disease recurrence.

Supplementary Table 3: Gene expression assays included into custom design TLDA card and used in single gene experiments

Assay ID	TLDA assays	Single gene assays	Gene symbol	Gene name
Hs01060665_g1	+	-	*ACTB*	actin, beta
Hs00984230_m1	+	-	*B2M*	beta-2-microglobulin
Hs00970220_m1	+	-	*CHI3L2*	chitinase 3-like 2
Hs01554629_m1	+	+	*ERG*	v-ets erythroblastosis virus E26 oncogene homolog (avian)
Hs00544833_m1	+	+	*EZH2*	enhancer of zeste homolog 2 (Drosophila)
Hs00361426_m1	+	-	*FABP7*	fatty acid binding protein 7, brain
Hs99999905_m1	+	-	*GAPDH*	glyceraldehyde-3-phosphate dehydrogenase
Hs00184139_m1	+	-	*GHRH*	growth hormone releasing hormone
Hs00271672_s1	+	+	*GPR52*	G protein-coupled receptor 52
Hs00939627_m1	+	+	*GUSB*	glucuronidase, beta
Hs01056332_m1	+	+	*HPN*	hepsin
Hs02800695_m1	+	+	*HPRT1*	hypoxanthine phosphoribosyltransferase 1
Hs01938284_g1	+	+	*MT1E*	metallothionein 1E
Hs01552593_m1	+	+	*OLR1*	oxidized low density lipoprotein (lectin-like) receptor 1
Hs01667582_m1	+	-	*SAA2*	serum amyloid A2
Hs00972656_m1	+	+	*TERT*	telomerase reverse transcriptase
